# Supplementary material for: Relationship between Urine Creatinine and Urine Osmolality in Spot Samples among Men and Women in the Danish Diet Cancer and Health Cohort
Source: Toxics. 2021 Nov 1;9(11):282. doi: 10.3390/toxics9110282 (PMC8625939; doi:10.3390/toxics9110282)
Supplement: Supplementary file 1 [file toxics-09-00282-s001.zip › toxics-1435438 SPM.pdf]

toxics

MDPI

Supplementary Materials: Relationship between Urine Creatinine and Urine Osmolality in Spot Samples among Men and Women in the Danish Diet Cancer and Health Cohort

Selinay Ozdemir, Clara G. Sears, James M. Harrington, Aslak Harbo Poulsen, Jessie Buckley, Chanelle J. Howe, Katherine A. James, Anne Tjonneland, Gregory A. Wellenius, Ole Raaschou-Nielsen and Jaymie Meliker

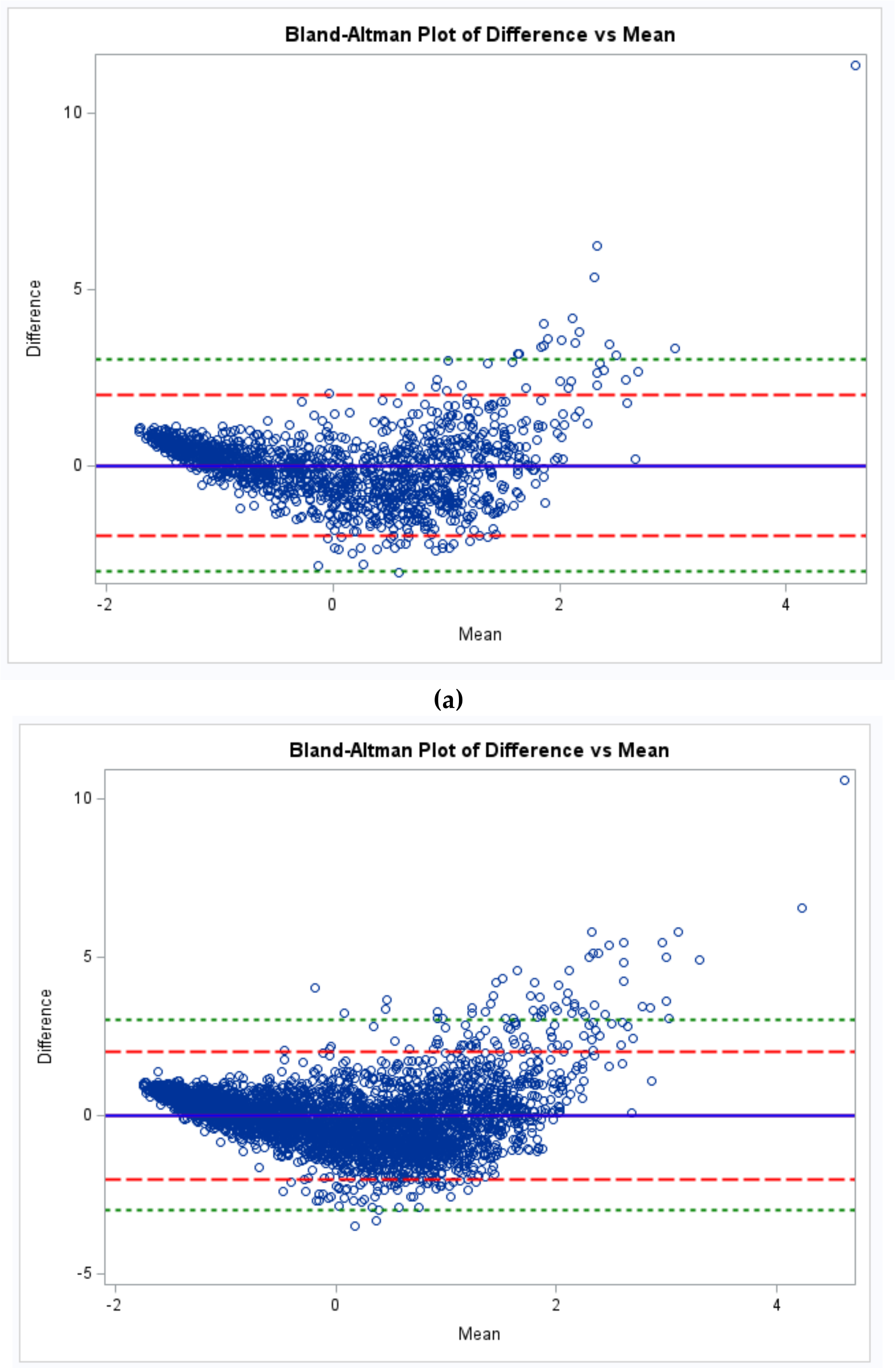

(a)

(b)

Figure S1. Bland-Altman Plot of the Difference between Osmolality and Creatinine Vs the Mean of Osmolality and Creatinine. Standardized with Z-Scores. (a) Subcohort. (b) Case-Cohort Study Population.

As the mean of creatinine and osmolality increases, the difference between the two values also increases, suggesting less similarity between the values at higher values of creatinine and osmolality.

Table S1. Characteristics of the Case-Cohort Study Population.

	N (%)	Median, 25th–75th %iles: Cr, mg/L	Median, 25th–75th %iles: Osmolality, mOsm
Case-Cohort Study Sample			
All	3731 (100)	1020 (481–1650)	591 (327–788)
Men	1991 (53)	1330 (764–1890)	690.5 (440–848)
Women	1740 (47)	685 (326–1280)	448 (247–699)
Age 50–<60	2525 (68)	1050 (504–1670)	610 (343–802)
Age 60–64	1206 (32)	948 (423–1590)	551 (299–759)
Diabetes at baseline	129 (3)	891 (426–1370)	643 (443–779)
Current Smoker	683 (18)	981 (461–1700)	550 (304–768)
Never Smoker	3048 (81)	1020 (487–1640)	602 (335–794)
BMI < 25	1306 (35)	865 (378–1470)	499 (265–746)
BMI 25 –30	1610 (43)	1090 (526–1700)	620 (354–799)
BMI ≥ 30	815 (22)	1150 (573–1720)	659 (402–824)
Incident Case Population			
AMI thru 2015	985 (26)	1080 (530–1680)	615 (355–808)
HF thru 2015	1135 (30)	1030 (480–1650)	610 (340–783)
Stroke thru 2009	709 (19)	1020 (480–1630)	567 (335–782)
Diabetes thru 2012	814 (22)	1090 (570–1680)	658 (395–814)

Table S2. Correlation coefficients between creatinine and osmolality in the subcohort and the case-cohort study sample.

	Subcohort		Case-cohort study sample	
	Spearman coefficient (ϱ), 95% CIs	Pearson coefficient (r), 95% CIs	Spearman coefficient (ϱ), 95% CIs	Pearson coefficient (r), 95% Cis
All	0.90 (0.89–0.91)	0.82 (0.80–0.84)	0.87 (0.87–0.88)	0.79 (0.78–0.80)
Men	0.84 (0.81–0.86)	0.79 (0.77–0.82)	0.82 (0.81–0.84)	0.77 (0.75–0.78)
Women	0.92 (0.91–0.93)	0.81 (0.78–0.83)	0.90 (0.89–0.90)	0.79 (0.77–0.80)
Age 50–<60	0.90 (0.89–0.91)	0.82 (0.80–0.84)	0.87 (0.86–0.88)	0.79 (0.78–0.81)
Age 60–64	0.90 (0.87–0.92)	0.81 (0.77–0.84)	0.88 (0.86–0.89)	0.80 (0.78–0.82)
Diabetes at Baseline	0.65 (0.27–0.85)	0.74 (0.43–0.89)	0.73 (0.63–0.80)	0.66 (0.55–0.75)
No Diabetes at Baseline	0.90 (0.89–0.91)	0.82 (0.80–0.84)	0.88 (0.87–0.89)	0.80 (0.79–0.81)
Current Smoker	0.88 (0.84–0.91)	0.81 (0.75–0.85)	0.86 (0.84–0.88	0.77 (0.73–0.79)
Never Smoker	0.90 (0.89–0.91)	0.82 (0.81–0.84)	0.88 (0.87–0.89)	0.81 (0.79–0.82)
BMI < 25	0.93 (0.91–0.94)	0.86 (0.84–0.88)	0.90 (0.89–0.91)	0.83 (0.81–0.84)
BMI 25–30	0.88 (0.86–0.90)	0.83 (0.81–0.86)	0.86 (0.85–0.88)	0.79 (0.77–0.81)
BMI > 30	0.83 (0.77–0.86)	0.70 (0.63–0.76)	0.83 (0.80–0.85)	0.74 (0.71–0.77)
Incident Case Populations				
AMI thru 2015	0.89 (0.82–0.93)	0.83 (0.73–0.89)	0.86 (0.84–0.88)	0.79 (0.76–0.81)
HF thru 2015	0.78 (0.66–0.86)	0.75 (0.62–0.84)	0.86 (0.84–0.87)	0.78 (0.75–0.80)
Stroke thru 2009	0.81 (0.68–0.89)	0.72 (0.55–0.84)	0.86 (0.84–0.88)	0.77 (0.74–0.80)
Diabetes thru 2012	0.78 (0.72–0.83)	0.77 (0.71–0.82)	0.79 (0.76–0.81)	0.72 (0.69–0.75)

95% CIs using Fisher's Z transformation

Table S3. Model R^2, Mean Square Error, and β coefficients of predictor variables selected in lasso procedure in relation to urine osmolality (mOsm) in case-cohort study sample.

	**B coefficient#**	**Mean Square Error**	**R^2**
Model 1 and Model 2*		26,721	0.63
Urine Creatinine (*mg*/L)	0.26		
Model 3		22,887	0.69
Urine Creatinine (*mg*/L)	0.17		
Urine Strontium (*µg*/L)	0.17		
Urine Cesium (*µg*/L)	12.98		
Urine Thallium (*µg*/L)	277.59		

*Results from Model 2 only showed creatinine associated with osmolality.

#These β coefficients indicate the change in osmolality per 1 unit increase in the predictor variable. The middle 50th percentile values for Sr were 116–336 *µg*/L, for Cs were 2.38–6.66 *µg*/L, and for Tl were 0.10–0.27 *µg*/L.

Table S4. Distributions and coefficients of variation of trace elements (*µg*/L) considered in the regression analysis.

	**Median**	**25–75 %iles**	**CV**
Co	0.45	0.26–0.67	9.8%
Zn	287	131–640	8.0%
As	21	10–46	7.0%
Se	22	12–38	9.6%
Sr	211	129–297	5.1%
Mo	31	13–58	4.6%
Cd	0.19	0.08–0.37	6.6%
Sn	0.42	0.20–0.84	16.5%
Sb	0.09	0.06–0.17	5.3%
Cs	4.2	2.5–7.0	6.4%
Ba	2.2	1.3–3.7	33.1%
Hg	0.49	0.06–1.44	11.5%
Tl	0.17	0.09–0.25	8.9%
Pb	1.26	0.72–2.32	9.6%

An iCAP Q ICP-MS system (Thermo Scientific, Waltham, MA) equipped with a helium gas collision cell was used for the determination of elements as described [19,20].
